# A high-resolution gene expression atlas of epistasis between gene-specific transcription factors exposes potential mechanisms for genetic interactions

**DOI:** 10.1186/s12915-015-0222-5

**Published:** 2015-12-23

**Authors:** Katrin Sameith, Saman Amini, Marian J. A. Groot Koerkamp, Dik van Leenen, Mariel Brok, Nathalie Brabers, Philip Lijnzaad, Sander R. van Hooff, Joris J. Benschop, Tineke L. Lenstra, Eva Apweiler, Sake van Wageningen, Berend Snel, Frank C. P. Holstege, Patrick Kemmeren

**Affiliations:** Molecular Cancer Research, University Medical Centre Utrecht, Universiteitsweg 100, Utrecht, The Netherlands; Theoretical Biology and Bioinformatics, Department of Biology, Utrecht University, Padualaan 8, Utrecht, The Netherlands

**Keywords:** Genetic interactions, Molecular mechanisms, Gene-specific transcription factors, *Saccharomyces cerevisiae*

## Abstract

**Background:**

Genetic interactions, or non-additive effects between genes, play a crucial role in many cellular processes and disease. Which mechanisms underlie these genetic interactions has hardly been characterized. Understanding the molecular basis of genetic interactions is crucial in deciphering pathway organization and understanding the relationship between genotype, phenotype and disease.

**Results:**

To investigate the nature of genetic interactions between gene-specific transcription factors (GSTFs) in *Saccharomyces cerevisiae*, we systematically analyzed 72 GSTF pairs by gene expression profiling double and single deletion mutants. These pairs were selected through previously published growth-based genetic interactions as well as through similarity in DNA binding properties. The result is a high-resolution atlas of gene expression-based genetic interactions that provides systems-level insight into GSTF epistasis. The atlas confirms known genetic interactions and exposes new ones. Importantly, the data can be used to investigate mechanisms that underlie individual genetic interactions. Two molecular mechanisms are proposed, “buffering by induced dependency” and “alleviation by derepression”.

**Conclusions:**

These mechanisms indicate how negative genetic interactions can occur between seemingly unrelated parallel pathways and how positive genetic interactions can indirectly expose parallel rather than same-pathway relationships. The focus on GSTFs is important for understanding the transcription regulatory network of yeast as it uncovers details behind many redundancy relationships, some of which are completely new. In addition, the study provides general insight into the complex nature of epistasis and proposes mechanistic models for genetic interactions, the majority of which do not fall into easily recognizable within- or between-pathway relationships.

**Electronic supplementary material:**

The online version of this article (doi:10.1186/s12915-015-0222-5) contains supplementary material, which is available to authorized users.

## Background

Predicting the phenotype of an individual organism based on its genotype is a major challenge. Such relationships can be complex, also because individual alleles can produce unexpected phenotypes in combination. The term epistasis has been used in distinct ways by classical and population geneticists [1, 2]. Epistasis was first introduced by Bateson to refer to the masking of one mutation by another [1]. Later, the term epistasis was generalized by Fisher to any non-additive genetic interaction whereby the combination of two mutations yields a phenotype that is unexpected based on the effect of the respective individual mutations [2]. Throughout the article, epistasis refers to the general definition by Fisher and is used synonymously with genetic interaction to refer to any unanticipated combinatorial effect.

Cell growth has frequently been used to study genetic interactions on a large scale [3–12]. Genetic interactions are measured by the extent to which growth defects of double deletion mutants deviate from their expected value. A widely applied model assumes that the expected double mutant fitness should be equivalent to the product of the two single mutants [13]. Genetic interactions scored by the difference between observed and expected fitness can broadly be classified into two groups: negative and positive genetic interactions. A genetic interaction is negative if the fitness observed for a double mutant is worse than expected based on the fitness of the respective single mutants. Conversely, an interaction is positive if the observed fitness is better than expected. The largest study available to date investigated the existence of genetic interactions for 5.4 million gene pairs in the model eukaryote *Saccharomyces cerevisiae*, identifying approximately 170,000 interactions [12]. The extent and pervasiveness of genetic interactions is evident from this study. Understanding genetic interactions and the mechanisms underlying them are therefore of obvious importance for understanding genotype-phenotype relationships.

Several mechanisms have been proposed for genetic interactions (reviewed in [14, 15]). The most intuitive explanation for a negative genetic interaction is redundancy, where two genes can substitute for one another by their ability to take over the exact same function [16]. Only simultaneous deletion of both genes has an effect on that function. A second explanation for a negative genetic interaction extends the concept of redundancy from genes to molecular pathways that function in parallel [14, 15]. Positive genetic interactions have been suggested to occur more often between genes functioning in the same pathway or complex [4, 17, 18]. Deletion of one gene causes dysfunction of the entire pathway or complex such that deletion of a second gene in the same pathway or complex has no further consequence. Although the interpretation of negative and positive genetic interactions as relationships between and within pathways is appealing, it leaves large parts of the epistatic landscape completely unexplained. First, duplicated, redundant genes only explain a small subset of negative genetic interactions [19, 20]. Second, many negative genetic interactions are detected between seemingly unrelated rather than parallel pathways [5, 6, 12]. And third, the vast majority of positive genetic interactions occur between genes encoding proteins in different pathways or complexes rather than the same [5, 12]. A theoretical model, “induced essentiality”, has been proposed in the past [21] that provides an explanation of how negative genetic interactions can occur between unrelated pathways. In this particular model, inactivation of one process results in an alternative condition that requires activation of another process. It does, however, leave non-essential genetic interactions unexplained and also lacks experimental data. Taken together, the molecular mechanisms underlying most genetic interactions are poorly characterized and further investigation is needed to provide a better mechanistic understanding of genetic interactions.

Transcription plays a major role in the relationship between genotype and phenotype. Depending on the state or fate of a cell, different genes are expressed at different levels. This is in part mediated by gene-specific transcription factors (GSTFs). Understanding the basis of genetic interactions between GSTFs is therefore likely to be important for understanding the transcription regulatory network. To study genetic interactions between GSTFs, genome-wide gene expression was monitored for 72 GSTF double deletion mutants and their corresponding single mutants. Gene expression has previously proven useful to study genetic interactions in more detail than is possible through growth defects [22–25]. The high-resolution expression atlas generated here provides a systems-level overview of the epistatic landscape between GSTFs and reveals underlying mechanistic details. Besides revealing new redundancy relationships, this study also proposes two molecular mechanisms. These mechanisms, which we term “buffering by induced dependency” and “alleviation by derepression”, provide explanations for negative and positive genetic interactions that were previously not understood.

## Results

### Growth-based genetic interaction scores

A genetic interaction between two genes can be studied by different phenotypes, of which cell growth is most frequently used. Here, growth is used in combination with genome-wide gene expression to investigate genetic interactions between GSTFs. *S. cerevisiae* has an estimated 215 GSTFs (Additional file 1; Methods). Selection of GSTF pairs likely having a genetic interaction is based on two distinct criteria. Pairs with a significant growth-based genetic interaction score as determined from a previous large-scale study [11] (47 pairs) and/or with similarity in their DNA binding properties [26–28] (50 pairs; Methods) were selected, resulting in a total of 90 pairs. Fitness of each deletion mutant is defined by its growth rate during exponential growth relative to wildtype (WT). Replicate relative growth rates (RGRs) are highly reproducible and were averaged for subsequent analyses (Additional file 2A; single mutants: R = 0.96, *P* <2.23 × 10^−308^; double mutants: R = 0.98, *P* <2.23 × 10^−308^). To score the genetic interaction ε_*growth,XY*_ between two GSTFs *X* and *Y*, fitness observed for the respective double mutant W_*xΔyΔ*_ is compared to the fitness that is expected based on both single mutants W_*xΔ*_ × W_*yΔ*_ (ε_*growth*,*XY*_ = W_*xΔyΔ*_ − W_*xΔ*_ × W_*yΔ*_) [13]. The resulting genetic interaction scores largely agree with the initial scores used for selecting GSTF pairs [27] (Additional file 2B; R = 0.63, *P* = 2.64 × 10^−5^), taking into account differences in the growth procedures (liquid culture versus agar plates) and media used (synthetic complete (SC) versus yeast extract peptone dextrose (YEPD)).

### Gene expression profiles of GSTF single and double deletion mutants

To investigate mechanisms of genetic interactions between GSTFs, gene expression profiles were generated for 154 single and double GSTF deletion mutants. WT cultures were grown and profiled alongside deletion mutants on each day to control for biological and technical variation. With the exception of single mutants that behave like WT, each mutant was grown and profiled four times from two independent cultures. Statistical modelling of the data results in an average expression profile for each mutant consisting of *P* values and fold changes (FC) for each gene relative to the average expression in a collection of WT cultures (Methods). Gene expression profiles for 72 GSTF pairs and their corresponding single deletion mutants successfully passed all quality controls and were used for further analysis (Additional file 1). These profiles provide the basis for a high-resolution atlas of genetic interactions between GSTFs.

### A gene expression atlas of epistasis

**Fig. 1:**
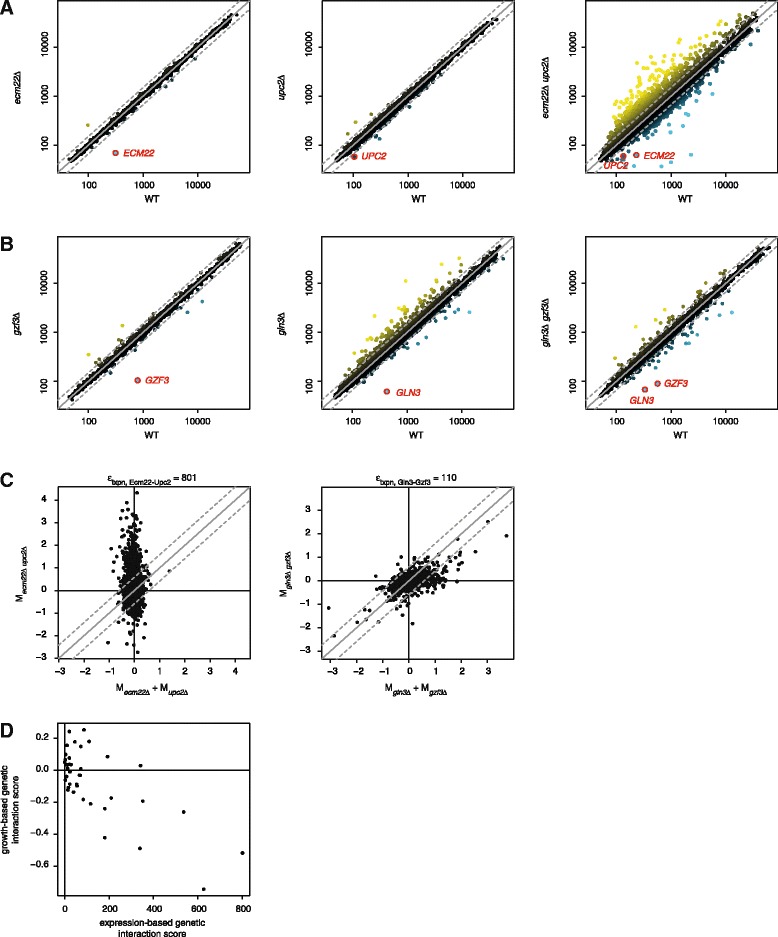
Genetic interactions measured by gene expression. **a** Genome-wide expression levels in the GSTF deletion mutants *emc22*Δ, *upc2*Δ and *ecm22*Δ *upc2*Δ (vertical, from left to right) versus reference WT (horizontal). Individual genes are depicted by solid circles, deleted genes are highlighted in red. Color range from yellow for increased expression levels relative to WT, black for unchanged expression, blue for decreased expression. A FC of 1.5 is depicted by a dashed grey line. **b** Genome-wide expression levels in the GSTF deletion mutants *gzf3*Δ, *gln3*Δ and *gln3*Δ *gzf3*Δ versus reference WT. Representation as in a. **c** Expected expression changes (M) in the double mutants *ecm22*Δ *upc2*Δ (horizontal, left panel) and *gln3*Δ *gzf3*Δ (right panel) versus observed expression changes (vertical). Individual genes are depicted by solid circles. Expected expression changes are calculated as the sum of expression changes in the respective single mutants. A FC of 1.5 is depicted by a dashed grey line. The number of genes outside the FC threshold is stated above each scatterplot. **d** Growth-based genetic interaction scores (vertical; liquid culture growth) are plotted versus expression-based genetic interaction scores (horizontal). Individual GSTF pairs are depicted by solid circles

A well appreciated mechanism of genetic interactions is complete redundancy [16], where two proteins can substitute for one another. For example, the two GSTFs Ecm22 and Upc2 redundantly activate sterol biosynthesis genes [29]. Deletion of either *ECM22* or *UPC2* does not affect growth and neither induces many expression changes (RGR_*ecm22Δ*_ = 1 and RGR_*upc2Δ*_ = 1.03; Fig. 1a, left and middle panel). Loss of one GSTF can almost completely be compensated by the presence of the second GSTF. Simultaneous deletion of both GSTFs, however, results in slow growth and many gene expression changes (RGR_*ecm22Δ upc2Δ*_ = 0.5; Fig. 1a, right panel). In addition to redundancy, other types of genetic interactions are exposed, for example between the two GSTFs Gzf3 and Gln3 involved in nitrogen regulation [30, 31]. Whereas deletion of *GZF3* has no effect on growth and expression (RGR_*gzf3Δ*_ = 1.02; Fig. 1b, left panel), deletion of *GLN3* results in a growth defect and many expression changes (RGR_*gln3Δ*_ = 0.8; Fig. 1b, middle panel). Intriguingly, these effects are suppressed in the double mutant (RGR_*gln3Δ gzf3Δ*_ = 0.99; Fig. 1b, right panel). These two examples show that both positive and negative genetic interactions can be studied using gene expression and that the observed expression changes may also be indicative of the genetic interaction type.

A potential advantage of investigating genetic interactions using gene expression is that changes can be compared at the level of individual genes. The effect of a genetic interaction between two GSTFs *X* and *Y* on a gene *i* can be measured as the deviation between the expression change observed in the double mutant M_*xΔyΔi*_ and the expected expression change, given each single mutant M_*xΔi*_ + M_*yΔi*_ (ε_*txpn,Xyi*_ = |M_*xΔyΔI*_ − (M_*xΔi*_ + M_*yΔi*_)|). If gene *i* is unaffected, observed and expected expression changes will be highly similar such that ε_*txpn,XYi*_ is close to zero. In other words, the stronger gene *i* is affected, the more ε_*txpn,XYi*_ deviates from zero. The genetic interaction between the GSTFs *X* and *Y* is then scored by counting the total number of genes for which an unexpected expression change can be observed in the respective double mutant. A FC of 1.5 is chosen as a threshold (ε_*txpn,XY*_ = ∑_*all genes i*_*f(i)*, with *f(i)* = 1, if ε_*txpn,Xyi*_ > log_2_(1.5); 0, else). Based on this threshold, the genetic interaction between Ecm22 and Upc2 involves 801 genes (Fig. 1c, left panel), the genetic interaction between Gln3 and Gzf3 involves 110 genes (Fig. 1c, right panel). With a few exceptions, genetic interaction scores derived by growth or expression are highly related (Fig. 1d; R = 0.75, *P* = 5.9 × 10^−8^, using absolute values for growth-based scores). GSTF double mutants that grow unexpectedly slow or fast often also show unexpected expression changes. However, as is exemplified below, expression-based genetic interaction scores provide a higher level of detail compared to growth-based genetic interaction scores and can be further used to investigate the underlying mechanisms of genetic interactions.

### Different expression patterns define the epistatic landscape

**Fig. 2:**
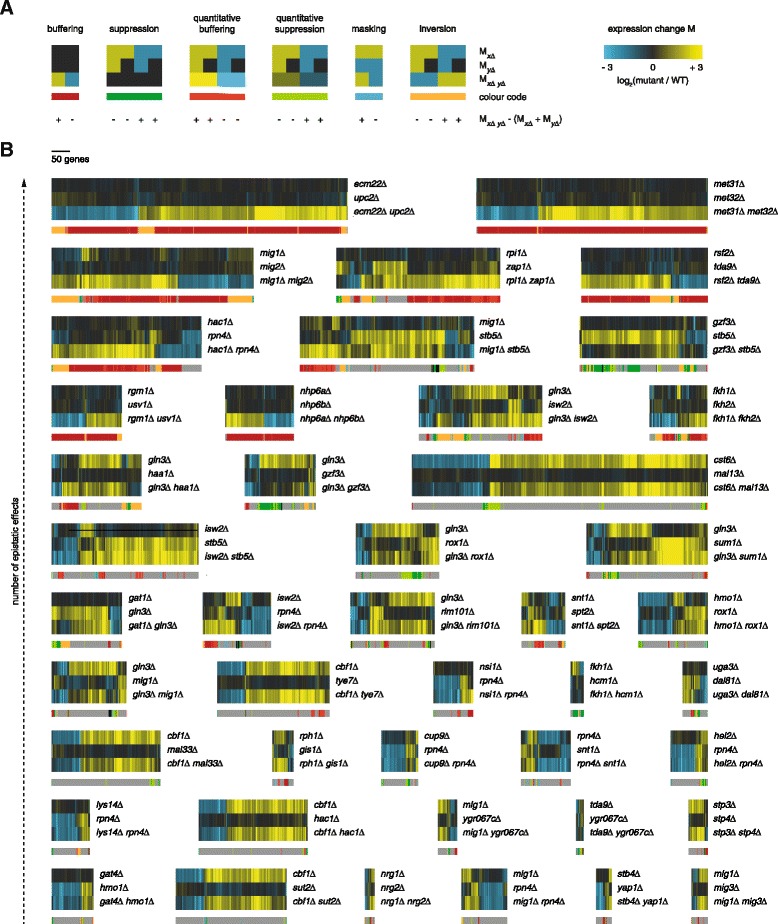
Gene expression atlas of GSTF pairs. **a** Cartoon of expression changes (horizontal) in GSTF single and double mutants (vertical). Color range from yellow for increased expression (*P* ≤0.01, FC >0), black for unchanged expression (*P* >0.01) and blue for decreased expression (*P* ≤0.01, FC <0), as depicted in the top-right corner. Types of epistatic effects are color-coded, shown below the cartoon expression data. At the bottom, it is stated for each expression pattern whether the observed expression change (M_*xΔ xΔ*_) is more positive (+) or more negative (−) than expected (M_*xΔ*_ + M_*yΔ*_). **b** Expression changes (horizontal) in GSTF single and double mutants (vertical). Color range as in a. Types of epistatic effects on individual genes are depicted below the expression changes (colors as in a; grey depicts non-epistatic expression changes). GSTF pairs are sorted according to the amount of epistatic effects (increasing from bottom to top). GSTF pairs with less than ten epistatic expression changes are not shown

The expression of individual genes can be affected by a genetic interaction between two GSTFs in several ways. In response to deletion of a single GSTF, expression of a gene can be decreased (*P* ≤0.01, FC <1), unchanged (*P* >0.01) or increased (*P* ≤0.01, FC >1), relative to WT. When comparing expression changes in two GSTF single mutants and their corresponding double mutant, for example *emc22Δ* and *upc2Δ*, and accounting for quantitative effects as well, 20 different expression patterns are observed that can be divided into six different types (Fig. 2a). The most intuitive expression pattern is buffering, where individual deletion of either GSTF does not affect expression, but simultaneous deletion results in many changes, including increased and/or decreased expression levels. Suppression is observed when expression changes elicited by one single mutant are suppressed by deletion of a second GSTF. Quantitative buffering is defined by expression changes induced by one single mutant that are amplified by deletion of a second GSTF. In contrast, quantitative suppression is observed if expression changes elicited by one GSTF single mutant are dampened by additional deletion of a second GSTF. Masking takes place if expression of a gene is increased (or decreased) in one single mutant, but this expression change is masked by decreased (or increased) expression in response to deletion of a second GSTF. Last, inversion is observed if expression of a gene is increased (or decreased) by individual deletion of either GSTF single mutant, but decreased (or increased) upon deletion of both GSTFs. Based on the different epistatic effects observed, an epistasis profile can be derived for all GSTF pairs.

With this approach, an atlas can be built consisting of the epistatic effects on expression levels by any two GSTFs under investigation (Fig. 2b). The number of individual genes showing expression-based genetic interactions varies throughout the GSTF pairs. It is immediately noticeable that buffering is predominant in epistasis profiles of many GSTF pairs (dark-red color in Fig. 2b), as expected based on the fact that genetic interactions are investigated between a single functional class of proteins (GSTFs). The abundance of buffering effects holds particularly for GSTF pairs with strong genetic interactions, affecting expression of many genes (Fig. 2b, top). Classification of expression patterns in GSTF single and double mutants facilitates an abstract view on the epistatic landscape that can be further harnessed to reveal different types of genetic interactions.

### Expression patterns expose the nature of genetic interactions

**Fig. 3:**
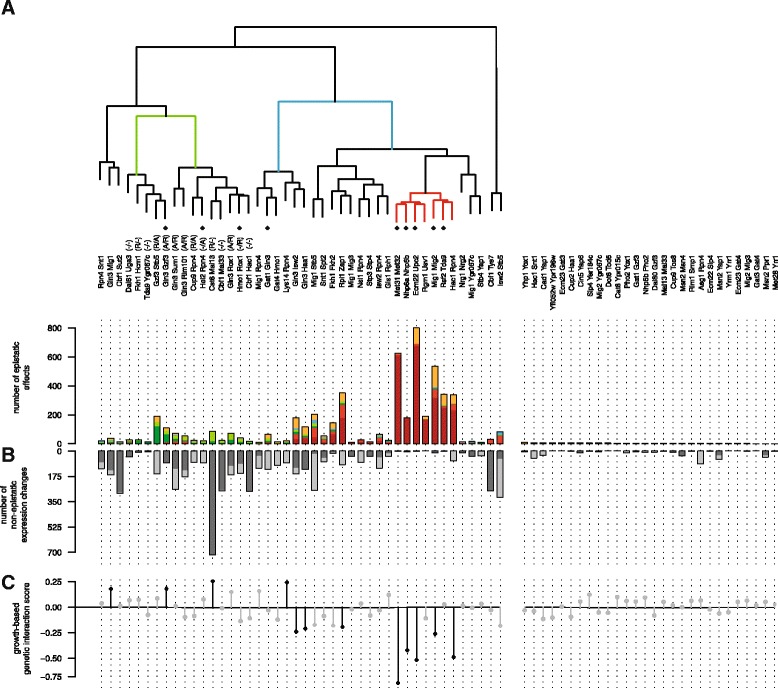
GSTFs have different genetic interactions. **a** Hierarchical clustering of GSTF pairs based on their epistatic effects. Average linkage clustering was applied to group GSTF pairs with similar epistatic effects. The identity of genes was disregarded. Instead, the number of total occurrences for each of the six different epistatic effects (Fig. 2a) were used. Similarities between GSTF pairs were calculated based on cosine correlation. Colored branches depict example groups described in the text. GSTFs marked with a diamond have DNA binding domains of the same type. The number of epistatic effects underlying the clustering are shown as bar-plots below the dendrogram (colors as in Fig. 2a). GSTF pairs with epistatic effects on less than ten genes are not included in the clustering, but are shown on the right. **b** Number of non-epistatic expression changes. Dark grey for the first named GSTF, light grey for the second. Counted are all genes with significantly changed expression (*P* ≤0.01) above a FC of 1.5. Left-to-right ordering as in a. **c** Growth-based genetic interaction scores depicted by solid circles. Significant scores are visualized in black, grey otherwise. Vertical lines for visual purpose only. Left-to-right ordering as in a

Hierarchical clustering was applied to group GSTF pairs with similar genetic interactions (Fig. 3a). Here, the identity of individual genes was disregarded. Instead, GSTF pairs with similar patterns of epistatic effects are clustered together. GSTF pairs separate into several distinct groups, indicating that they have different types of genetic interactions. GSTF pairs clustered on the left are generally characterized by suppressive effects (Fig. 3a, green branch), whereas GSTF pairs clustered on the right are generally characterized by buffering effects (Fig. 3a, blue branch). From this analysis it is clear that buffering effects appear to be a good predictor for slow growth and vice versa (Fig. 3b,c). If expression is changed only upon deletion of two GSTFs, the respective double mutant often grows slower than expected (negative interaction, e.g. Ecm22-Upc2). In turn, if expression changes induced by deletion of one GSTF are suppressed by additional deletion of the second GSTF, the respective double mutant sometimes grows faster than expected (positive genetic interaction, e.g. Gln3-Gzf3).

To investigate the difference between selecting GSTF pairs either on growth-based genetic interactions or similarity in DNA binding, hierarchical clustering was applied to both groups individually (Additional file 3). Several interesting differences emerge from this comparison. First, of all GSTF pairs selected based on growth (Additional file 3A) approximately 87 % (33 out of 38 pairs) show an expression-based genetic interaction. Only 17 of the 40 (43 %) GSTF pairs selected based on similarity in DNA binding (Additional file 3B) have an expression-based genetic interaction. The fact that individual as well as simultaneous deletion of the remaining 23 pairs hardly affects expression may in part be due to condition specificity. Two of the three selection criteria used for similarity in DNA binding are based on *in vitro* (promoter affinity scores) and *in silico* (similarity of DNA binding domain) criteria, with no evidence for functional relevance under the condition investigated. For example, Msn2 and Msn4 are two redundant GSTFs involved in stress response [32] and are therefore likely inactive in non-stress conditions as used in this study. Second, pairs selected based on similarity in DNA binding mostly show buffering effects (Additional file 3B, red branch) and only a few pairs are characterized by suppressive relationships (Additional file 3B, green branch). Pairs selected based on growth, on the other hand, show much more suppressive effects (Additional file 3A, green branch). These pairs also show both positive genetic interactions (Additional file 3A; four pairs) as well as negative genetic interactions (eight pairs), whereas pairs selected based on similarity in DNA binding are mostly showing negative genetic interactions (Additional file 3B; four pairs) and little positive genetic interactions (one pair). This all indicates that pairs selected based on similarity in DNA binding show a strong bias towards selecting redundant relationships, whereas pairs selected based on growth show a broader spectrum of genetic interaction types.

One group of six GSTF pairs is particularly distinctive (Fig. 3a, red branch). First, GSTF pairs in this group are strongly epistatic, with buffering effects on many genes (Fig. 3b, red bars). Second, six out of the seven GSTF pairs show very few expression changes that are not epistatic (Fig. 3b). Third, most pairs contain a DNA binding domain of the same type (Fig. 3a, diamonds). Together, these three characteristics are indicative of redundancy relationships. Indeed, redundancy relationships have previously been described for Ecm22-Upc2 [29], Met31-Met32 [33], Nhp6a-Nhp6b [34] and Mig1-Mig2 [35].

### Rgm1 and Usv1 redundantly activate genes involved in respiratory ATP synthesis

**Fig. 4:**
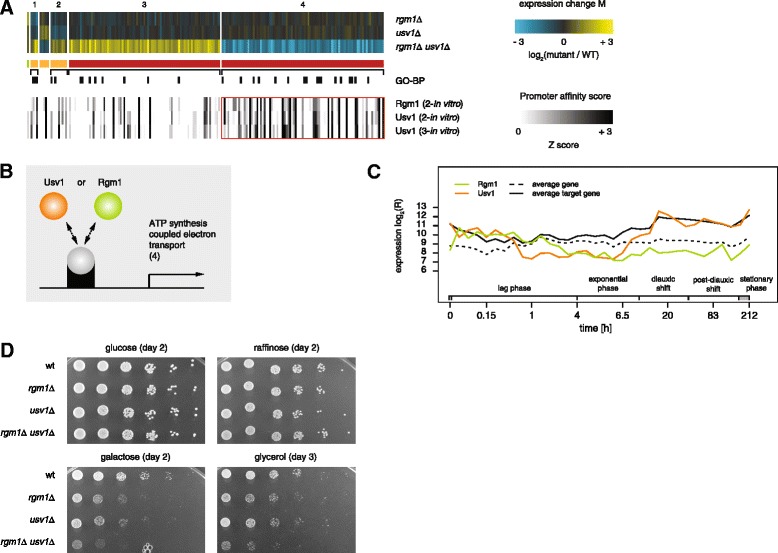
Rgm1 and Usv1 activate genes involved in respiratory ATP synthesis. **a** Genetic interaction between Rgm1 and Usv1. Co-expressed genes are separated into different sets based on increased, unchanged or decreased expression levels in the GSTF single and double mutants (top). Color scale as in Fig. 2a. Types of epistatic effects on individual genes are depicted below the expression changes (colors as in Fig. 2a). Gene sets are sorted by size. Annotations of the top GO-BP terms enriched in individual gene sets are presented below the expression data; gene set 1: monocarboxylic acid metabolic process (*P* = 3.64 × 10^−4^); gene set 2: agglutination involved in conjugation with cellular fusion (*P* = 3.3 × 10^−4^); gene set 3: iron ion transport (*P* = 2.03 × 10^−8^); gene set 4: ATP synthesis coupled electron transport (*P* = 1.69 × 10^−23^). Available DNA binding data showing significant overlap with at least one gene set are presented at the bottom. These are *in vitro*-derived promoter affinity scores (“2-*in vitro*”, “3-*in vitro*” as calculated from [28] and [27], respectively; Methods). Promoter affinity scores range from zero (white) to three (black) as depicted. Significant correspondence with expression data is depicted by red boxes. **b** Cartoon depicting the proposed genetic interaction between Rgm1 and Usv1. **c** Log-transformed expression levels of Rgm1 (green solid), Usv1 (orange solid), and average expression levels of their activated genes (black solid; gene set 4 in a) as well as all genes (black dashed line) throughout different growth phases [58]. **d** Spot assays showing growth on different carbon sources

Another interesting GSTF pair that clusters tightly with the redundant GSTF pairs Met31-Met32, Nhp6a-Nhp6b and Ecm22-Upc2 is Rgm1-Usv1 (Fig. 3c, red branch). Besides having a binding site of the same type, Rgm1 and Usv1 have thus far not been reported to genetically interact. Individual deletion of either *RGM1* or *USV1* has no consequences for growth and almost no consequences for expression (RGR_*rgm1Δ*_ = 0.99; RGR_*usv1Δ*_ = 1; Fig. 4a). Simultaneous deletion, on the other hand, results in slower growth and many expression changes (RGR_*rgm1Δ usv1Δ*_ = 0.89; Fig. 4a). Rgm1 and Usv1 contain a DNA binding domain of the same type (zinc finger pair [36]), and *in vitro*-derived promoter affinity scores of Rgm1 and Usv1 are highly correlated [27, 28] (R = 0.94; Methods). Binding affinities of both GSTFs are significantly increased for genes that are unaffected in the single mutants *rgm1Δ* and *usv1Δ*, but show decreased expression in the double mutant *rgm1Δ usv1Δ* (Fig. 4a, gene set 4). Rgm1 and Usv1 therefore likely activate transcription of these genes redundantly (Fig. 4b). The top functional category enriched among Rgm1- and Usv1-activated genes is “ATP synthesis coupled electron transport” (*P* = 1.69 × 10^−23^), a respiration-related process. Yeast cells preferentially produce energy through fermentation, but switch to respiration when fermentable carbon sources such as glucose are depleted [37, 38]. Rgm1 and Usv1 are probably also active at basal levels when glucose is available, since expression changes are measured during exponential fermentative growth. The expression levels of Usv1 and Rgm1 as well as their target genes are increased during growth phases that require respiration (Fig. 4c, lag phase, diauxic shift to stationary phase). Interestingly, the expression level of Rgm1 and Usv1 differs during the shift from fermentation to respiration, indicating that Rgm1 and Usv1 may not be completely redundant under these conditions (Fig. 4c, diauxic shift to stationary phase). This hypothesis is further supported by growth assays of *rgm1Δ, usv1Δ* and *rgm1Δ usv1Δ* on different carbon sources (Fig. 4d). If Rgm1 and Usv1 are completely redundant under any given growth condition, deletion of one of the two factors is not expected to affect growth. Indeed, no growth defect is visible for any mutant during growth on the fermentable carbon sources glucose and raffinose. On the other hand, during growth on galactose, a less preferred fermentable carbon source, as well as the non-fermentable source glycerol, the single mutants *rgm1Δ* and *usv1Δ* grow markedly slower than WT and this growth defect is amplified in the double mutant *rgm1Δ usv1Δ*. Taken together, these results provide evidence that Rgm1 and Usv1 act redundantly, at least under exponential growth on glucose, to activate genes involved in respiratory ATP synthesis.

### Buffering by induced dependency as a potential mechanism underlying negative genetic interactions

**Fig. 5:**
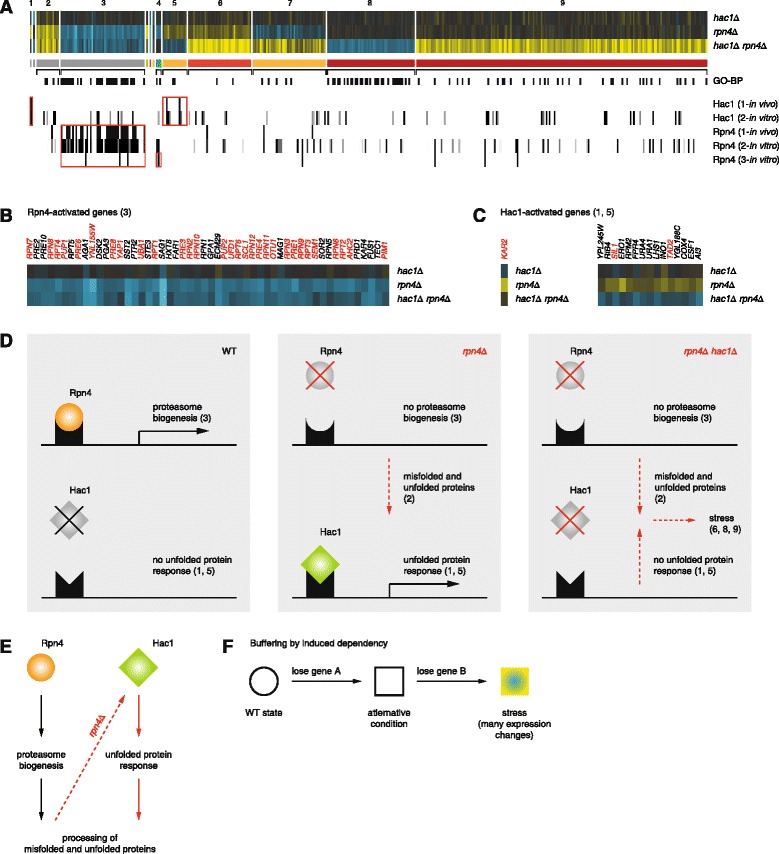
Buffering by induced dependency. **a** Genetic interaction between Hac1 and Rpn4. Representation as described for Fig. 4a. Grey depicts non-epistatic expression changes. Available DNA binding data showing significant overlap with at least one gene set are presented below the expression data. These are *in vivo* binding targets (“1-*in vivo*” as in [26]) as well as *in vitro*-derived promoter affinity scores (“2-*in vitro*”,“3-*in vitro*” as calculated from [28] and [27], respectively; Methods). Promoter affinity scores range from zero (white) to three (black) as depicted. Significant correspondence with expression data is depicted by red boxes. Top GO-BP terms are: gene set 2: protein refolding (*P* = 2.85 × 10^−6^); gene set 3: modification-dependent catabolic process (*P* = 1.29 × 10^−32^); gene set 4: transmembrane transport (*P* = 0.04); gene set 5: *de novo* pyrimidine nucleobase biosynthetic process (*P* = 9.79 × 10^−3^); gene set 6: response to water deprivation (*P* = 0.03); gene set 7: glutamine family amino acid biosynthetic process (*P* = 2.7 × 10^−4^); gene set 8: ribosome biogenesis (*P* = 1.55 × 10^−21^); gene set 9: oxidation-reduction process (*P* = 1.1 × 10^−6^). **b** Rpn4-activated genes (zoom-up of gene set 3 in a). Red labels for annotated target genes of Rpn4 *in vivo* [26]. **c** Hac1-activated genes (zoom-up of gene sets 1 and 5 in a). Red labels for annotated target genes of Hac1 *in vivo* [26]. **d** Cartoon depicting the proposed genetic interaction between Hac1 and Rpn4. Consequences of individual deletions are indicated in red. **e** Summarized model to describe the proposed genetic interaction between Hac1 and Rpn4. **f** Generalized model for “buffering by induced dependency”

Negative genetic interactions have often been associated with redundant genes and this is reflected by GSTF pairs such as Ecm22-Upc2 [29], Met31-Met32 [33] and Mig1-Mig2 [35]. Buffering can also occur between genes in parallel pathways that can compensate for each other’s loss [15]. A related mechanism termed “induced essentiality” has been proposed [21]. So far this has remained a theoretical model with no examples reported. Here, at least one GSTF pair potentially exhibits a closely related mechanism. In contrast to “induced essentiality”, the `potential mechanism observed here, does not lead to lethality, but instead results in a stress response. We therefore term this as “buffering by induced dependency”. Hac1 and Rpn4 activate transcription of genes involved in two different pathways that are linked to the processing of inappropriately folded proteins, the unfolded protein response [39] (UPR, Hac1) and the endoplasmic reticulum-associated degradation (ERAD) by the proteasome (Rpn4) [40]. Deletion of *HAC1* neither affects growth or expression (RGR_*hac1Δ*_ = 1.05; Fig. 5a), indicating that the UPR is inactive in WT (Fig. 5d, WT panel). Deletion of *RPN4*, on the other hand, induces a mild growth defect and results in decreased expression of its proteasomal target genes (RGR_*rpn4Δ*_ = 0.9; Fig. 5a, gene set 3; Fig. 5b). It further results in increased expression of Hac1 target genes including *KAR2* [26, 41] (Fig. 5c). This agrees with a previous observation that disruption of the ERAD pathway leads to activation of the UPR due to accumulation of misfolded and unfolded proteins in the endoplasmic reticulum [42] (Fig. 5d, *rpn4Δ* panel). Expression changes elicited by the double mutant *hac1Δ rpn4Δ* indicate that disruption of both the ERAD and UPR induces severe stress (Fig. 5d, *hac1Δ rpn4Δ* panel). Simultaneous repression of transcripts involved in translation (Fig. 5a, gene set 8) and induction of transcripts involved in respiration (Fig. 5a, gene set 9) are hallmarks of a stress response [43]. Moreover, selective and non-selective autophagy may be activated [44] (suggested by increased expression of the autophagy-related gene *ATG8*) coupled to vacuolar degradation [45] (suggested by increased expression of the peptidase PRC1 and proteinase PRB1). The activation of these processes may help the cells to survive, but are not sufficient to compensate the disruption of both ERAD and UPR as is reflected by a strong growth defect and many expression changes in the double mutant *hac1Δ rpn4Δ* (RGR_*hac1Δ rpn4Δ*_ = 0.45). Additional follow-up experimentation will have to be performed in order to further substantiate the proposed model. Taken together, the genetic interaction between Hac1 and Rpn4 suggests how two pathways can buffer each other in a non-redundant, non-essential manner, whereby one pathway is only required because the other pathway has been inactivated: buffering by induced dependency (Fig. 5e,f).

### Alleviation by derepression as a potential mechanism underlying positive genetic interactions

**Fig. 6:**
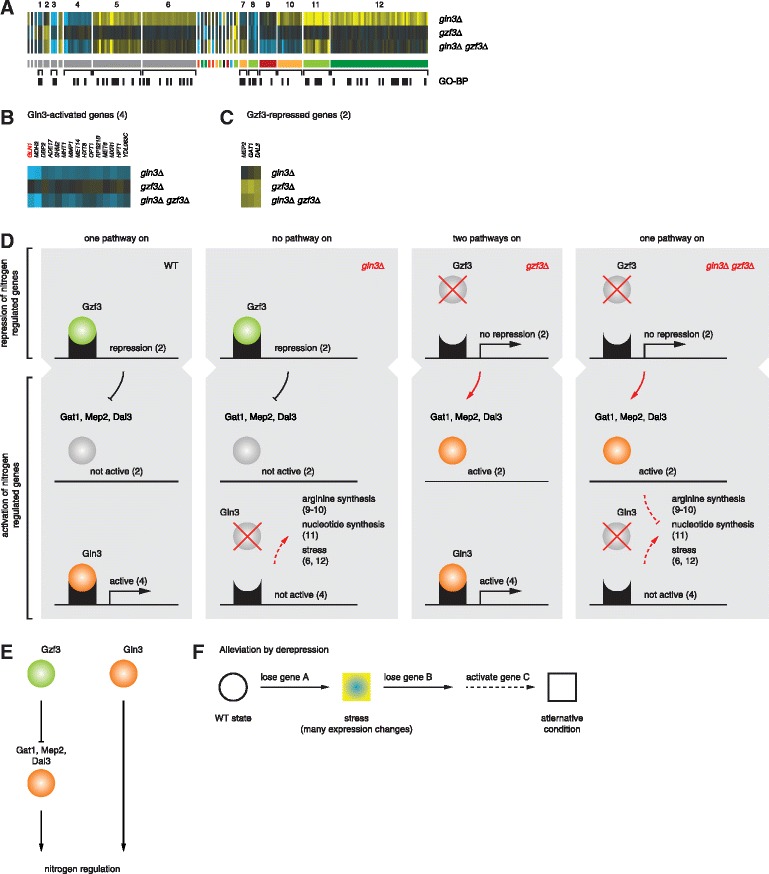
Alleviation by derepression. **a** Genetic interaction between Gln3 and Gzf3. Representation as described for Fig. 5a. Top GO-BP terms are: gene set 1: generation of precursor metabolites and energy (*P* = 5.86 × 10^−3^); gene set 3: *de novo* pyrimidine nucleobase biosynthetic process (*P* = 9.58 × 10^−5^); gene set 4: methionine biosynthetic process (*P* = 4.78 × 10^−3^); gene set 5: oxidation reduction process (*P* = 4.32 × 10^−4^); gene set 6: generation of precursor metabolites and energy (*P* = 1.09 × 10^−6^); gene set 7: oxidation reduction process (*P* = 0.01); gene set 8: response to pheromone involved in conjugation with cellular fusion (*P* = 3.12 × 10^−4^); gene set 9: arginine biosynthetic process (*P* = 4.44 × 10^−3^); gene set 10: arginine biosynthetic process (*P* = 5.45 × 10^−3^); gene set 11: nucleotide biosynthetic process (*P* = 1.11 × 10^−6^); gene set 12: oxidation reduction process (*P* = 5.28 × 10^−6^). **b** Gln3-activated genes (zoom-up of gene set 4 in a). Red label to highlight the known target gene *GLN1* [48]. **c** Gzf3-repressed genes (zoom-up of gene set 2 in a). **d** Cartoon depicting the proposed relationship between Gln3 and Gzf3. Consequences of individual deletions are indicated in red. **e** Summarized model to describe the proposed genetic interaction between Gln3 and Gzf3. **f** Generalized model for “alleviation by derepression”

Many GSTF pairs are characterized by suppressive effects in the double mutant (Figs. 2b and 3). For two pairs, these expression changes are also reflected in a positive growth-based genetic interaction (Fig. 3c). Positive genetic interactions have been suggested to occur more often between genes functioning in the same pathway or complex [4, 17, 18]. This leaves the majority of positive genetic interactions unexplained [15]. Similarly, same-pathway or same-complex relationships are not obvious or reported for the GSTF pairs with suppressive effects (Fig. 3b, green branch). Instead, all GSTFs within this branch, for which systematic classification into either activator or repressor was available [46], form activator-repressor pairs. We therefore focused on one of these pairs to propose a likely mechanism. Gln3 and Gzf3 are two of four closely related GATA GSTFs involved in different aspects of nitrogen catabolite repression, a process that prevents the production of enzymes and permeases for the utilization of non-preferred nitrogen sources when a preferred nitrogen source is available [47]. Transcription activation through Gln3 and Gat1 is counteracted by repression through Gzf3 and Dal80 [47]. Deletion of *GLN3* alone results in a growth defect and changed expression of many genes (RGR_*gln3Δ*_ = 0.8; Fig. 6a), including decreased expression of the well-known target gene *GLN1* [48] (Fig. 6b). Gln1 is an enzyme that synthesizes glutamine from glutamate and ammonium [49]. Arginine can serve as a source for glutamine synthesis and glutamine is an input into amino acid and nucleotide biosynthesis [47]. Increased activity of enzymes involved in these processes may help to compensate for lower amounts of available glutamine. Indeed, as a secondary response to the limited activity of Gln1, expression of genes involved in arginine biosynthesis as well as *de novo* nucleotide biosynthesis is increased (Fig. 6a, gene sets 10, 11; Fig. 6d, *gln3Δ* panel). In contrast to *gln3Δ*, deletion of *GZF3* has no effect on growth and results in very few expression changes (RGR_*gzf3Δ*_ = 1.02; Fig. 6a). Deletion of *GZF3* does, however, result in increased expression for a second activator Gat1 [50], as well as two other GATA regulated genes *MEP2* and *DAL3* [47, 51] (Fig. 6c; Fig. 6d, *gzf3Δ* panel). Deletion of *GZF3* in the genetic background of *gln3Δ* alleviates the growth defect and suppresses many of the expression changes observed upon deletion of *GLN3* alone (RGR_*gln3Δ gzf3Δ*_ = 0.99; Fig. 6a, gene sets 9–11 and gene sets 6, 12, respectively). A likely explanation is that derepression of Gat1, Mep2 and/or Dal3 in *gzf3Δ* can compensate for the loss of *GLN3* (Fig. 6d, *gln3Δ gzf3Δ* panel). Although additional evidence is needed to further validate the results, the example of Gln3 and Gzf3 suggests a molecular mechanism underlying positive genetic interactions. Within this mechanistic model, the effects of deleting one gene are alleviated by deleting a second gene, through derepression of a third gene (Fig. 6e–f, “alleviation by derepression”). Since many of the other pairs with suppression effects consist of an activator and a repressor, alleviation by derepression may also hold for other pathways, providing a mechanistic explanation for how positive genetic interactions can nevertheless occur between different pathways.

## Discussion

### Positive genetic interactions between parallel, non-redundant pathways

We systematically investigated the epistatic landscape between yeast GSTFs by monitoring genome-wide gene expression changes of single and double deletion mutants. As a resource, the generated expression atlas can be harnessed in several ways. In addition to providing insights into the epistatic network between yeast GSTFs, the atlas exposes mechanistic details for negative and positive genetic interactions.

A number of molecular mechanisms have been proposed to underlie positive genetic interactions. For example, it has been suggested that positive genetic interactions could occur more often between genes encoding proteins of the same pathway or complex [4, 17, 18]. The reasoning behind this expectation is that deletion of any individual gene will cause dysfunction of the entire pathway or complex, and thus deletion of a second gene will have no further consequence. Moreover, positive genetic interactions are suggested to occur in signaling pathways where two proteins have opposing influences on pathway activity. This (Batesonian) epistasis has been successfully applied to order genes in signaling pathways [22, 23]. However, it has been shown that positive genetic interactions occur more frequently between different complexes or pathways rather than the same [5, 12]. A molecular mechanism for such a positive genetic interaction is proposed in this study (“alleviation by derepression”), based on the epistatic effects observed for the two GSTFs Gln3 and Gzf3. The effects of inactivation of one gene/pathway may be buffered by the activation of another gene/pathway. However, that gene/pathway is repressed. Only upon deletion of the repressor can buffering take place and the effects of the single mutant are suppressed. In addition to Gln3-Gzf3, many other GSTF pairs with suppressive effects consist of an activator and a repressor. The molecular mechanism proposed for Gln3-Gzf3 is therefore likely not an exception and may be applicable to these pairs as well.

### Negative interactions between parallel, non-redundant pathways

The perhaps simplest cause of negative genetic interactions is genetic redundancy, where two genes can completely substitute for one another. The first systematic genetic interaction surveys, however, quickly revealed that genetic redundancy accounts for only a small subset of all negative genetic interactions [19, 20]. Extending the concept of redundancy to molecular pathways does not explain either why many negative interactions are observed between seemingly unrelated pathways [5, 12]. In this study, a molecular mechanism is proposed that may provide this missing link. The GSTF pair Hac1-Rpn4 alludes how a negative genetic interaction can be caused by the regulatory responses of a cell to deletion of a gene rather than by simple redundancy (“buffering by induced dependency”). The observed relationship is akin to a theoretical model (“induced essentiality”) that has previously been suggested [21] to explain many synthetic lethal interactions. The model introduced here is closely related. In both models, loss of one gene leads to a rearrangement into an alternative condition, where a second gene has become important for a particular cellular process. The key difference is that whereas in the “induced essentiality” model, the second gene has become essential, in our model this is not the case. It does, however, lead to (severe) stress when also removed as the cell is unable to appropriately cope with the resulting loss of function. The extent to which these mechanisms apply to other negative genetic interactions remains to be determined, but “buffering by induced dependency” proposes a mechanistic explanation for negative genetic interactions that cannot be explained by simple redundancy.

### Genome-wide expression as a tool to understand the nature of genetic interactions

The development of synthetic genetic arrays [52] alongside computational methods for data analysis [17] facilitated the study of genetic interactions in a high-throughput manner. On the basis of growth as a fitness measure, the epistatic landscape, both static and dynamic, has been quantified in several large-scale studies [3–12]. In addition to growth, genome-wide expression has been used to study the nature of genetic interactions. In a study of kinase/phosphatase genetic interactions [25] this increased resolution revealed “mixed epistasis”, whereby two yeast kinases and/or phosphatases exert different epistatic effects on different genes. Mixed epistasis is also observed between GSTFs, but to a lesser degree when compared to kinase/phosphatase pairs. This may be due to the fact that expression changes occur as direct consequences of the inactivity of a GSTF. Inactivity of a kinase or phosphatase first has to be communicated through a signaling pathway, offering additional possibilities for interconnectivity. As large-scale fitness studies continue to reveal the full spectrum of the epistatic network, the additional use of high-resolution phenotypes such as expression is beneficial to increase our understanding of the underlying molecular mechanisms. In turn, increased mechanistic understanding facilitates a better interpretation of present and future large-scale genetic interaction studies and elucidate complex genotype-to-phenotype relationships.

## Conclusions

Here, we have investigated the nature of genetic interactions between gene-specific transcription factors in baker’s yeast. Systematic analysis of 72 GSTF pairs results in a high-resolution atlas of gene expression-based genetic interactions. The atlas exposes both novel genetic interactions as well as confirms known ones. More importantly, the data is used to investigate mechanisms underlying genetic interactions. Two mechanisms, “buffering by induced dependency” and “alleviation by derepression”, are proposed. These mechanisms indicate how negative genetic interactions can occur between seemingly unrelated parallel pathways as well as how positive genetic interactions can indirectly expose parallel rather than same-pathway relationships. The study provides general insights into the complex nature of genetic interactions and proposes mechanistic models for genetic interactions that help us understand the full spectrum of genetic interactions and their contribution to cellular processes and pathway organization.

## Methods

### Selection of GSTF pairs

A list of 215 putative GSTFs was compiled based on 1) the presence of a DNA binding domain and 2) evidence for specific DNA binding (Additional file 1). For 12 putative GSTFs, the latter criterion was not fulfilled. These were included nevertheless, because they contain a domain that previously has been associated with specific DNA binding for another GSTF. Putative genetically interacting GSTF pairs were selected based on two distinct criteria. First, GSTF pairs were selected that exhibit significant genetic interactions based on growth [11]. Significance of a genetic interaction was estimated by z-transformation of the genetic interaction scores. A single genetic interaction score was compared to all other scores in the entire dataset (28 pairs), as well as to all other scores of one of the two GSTFs of interest (37 pairs; 47 pairs in total). Second, GSTF pairs were selected based on evidence for common DNA binding. These are GSTF pairs with similar DNA binding domains (19 pairs), GSTF pairs with common *in vivo* target genes [26] (ten pairs) and GSTF pairs with similar promoter affinity profiles calculated from *in vitro* data [27, 28] (30 pairs, 50 pairs in total for the second criterion). Altogether, 90 GSTF pairs were selected (Additional file 1).

### Yeast strains

All strains are isogenic to S288c. Single mutants (Additional file 1) were taken from the Saccharomyces Genome Deletion library and obtained from Euroscarf (Frankfurt, Germany) or Open Biosystems (Huntsville, AL, USA). Double mutants were generated in duplicate by haploid transformation, random spore analysis or tetrad dissection, in an identical genetic background as the single mutants (Additional file 1). All single mutants and most double mutants carry the mating type matα and are in the genetic background of BY4742. Few double mutants carry the mating type matA and are in the genetic background of BY4741. In six strains from the collection, gene expression profiles revealed different defects. Note that such defects may be common to all copies of the collection but could also have arisen due to our handling of these strains. All these strains were remade. Of the selected 90 double mutants, 18 failed quality control criteria even after remake. They were therefore excluded from further analyses. In total, 154 deletion mutants passed our quality control criteria (Additional file 1).

### Gene expression profiling

Full details of all gene expression profiling procedures have been described before [46]. In summary, deletion mutants were grown in rich medium (SC, supplemented with 2 % glucose) and harvested in early mid-log phase. WT cultures were grown alongside and processed in parallel. Dual-channel 70-mer oligonucleotide arrays were employed with WT RNA as common reference. All steps after RNA isolation were automated using robotic liquid handlers. These procedures were first optimized for accuracy (correct FC) and precision (reproducible result), using spiked-in RNA calibration [53]. After quality control, normalization and dye-bias correction [54], statistical analysis was performed for each mutant versus a collection of WT cultures [25, 46, 55]. Single mutants differing from WT as well as all double mutants were profiled another two times from an independently inoculated culture. The reported FC is then the average of four replicate mutant expression profiles versus the average of all WTs. Genes that show variable expression changes in the WT collection were excluded from further analyses (57 WT variable genes in total), as well as YDL196W.

### Growth-based genetic interaction scores

Strains were grown in a Tecan Infinite F200 microplate reader, an automated system to incubate and measure optical densities in microplates. Growth measurements were taken every ten minutes, until cells were harvested at an OD_600_ of 0.6. Measurements that fall into exponential growth phase were selected to calculate growth rates. These are generally between an OD_600_ of 0.3 and the last measurement at an OD_600_ of 0.6. Growth rates were calculated as the slope of the selected log-transformed measurements.

The fitness *W* of a deletion mutant was determined as the fraction between the average growth rate of WT and the growth rate of the mutant. Mutants growing slower than WT hence result in a fitness smaller than one. The genetic interaction ε_*growth,XY*_ between two GSTFs *X* and *Y* was scored by comparing the fitness of the respective double mutant *W*_*xΔyΔ*_ with the fitness expected based on both single mutants W_*xΔ*_ × W_*yΔ*_ (ε_*growth*,*XY*_ = W_*xΔyΔ*_ − W_*xΔ*_ × W_*yΔ*_) [13]. Significance of genetic interaction scores is derived by z-transformation. Background genetic interaction scores (100,000 in total) were calculated by randomly selecting triplets of WTs and applying the same calculation as applied to evaluate genetic interactions between GSTFs. Resulting *P* values were corrected for multiple testing using Benjamini-Hochberg; adjusted *P* values lower than 0.05 were considered significant. Fitness values of all single and double mutants, as well as calculated genetic interaction scores can be found in Additional file 4.

### Expression-based genetic interaction scores

For a given GSTF pair, only genes with a statistically significant expression change in at least one of the two respective single mutants or in the double mutant were considered (*P* ≤0.01). The epistatic effect of two GSTFs *X* and *Y* on the expression of a gene *i* was measured as the deviation between the expression change observed in the double mutant *M*_*xΔyΔi*_ and the expression change expected given the single mutants *M*_*xΔi*_ + *M*_*yΔi*_ (ε_*txpn*,*XYi*_ = |*M*_*xΔyΔi*_ − (*M*_*xΔi*_ + *M*_*yΔi*_)|). The overall genetic interaction between the GSTFs *X* and *Y* was then scored by counting the total number of genes for which an unexpected expression change can be observed in the respective double mutant, whereby an FC of 1.5 was chosen as a threshold (ε_*txpn,XY*_ = ∑_*all genes i*_*f(i)*, with *f(i)* = 1, if ε_txpn,XYi_ > log_2_(1.5); 0, else).

For each GSTF pair, genes with epistatic expression changes (all genes *i* where ε_*txpn,XYi*_ > log_2_(1.5)) were further divided into different sets based on the observed expression patterns. Depending on whether expression levels are increased relative to WT (*P* ≤0.01, FC >0), unchanged (*P* >0.01) or decreased (*P* ≤0.01, FC <0) in either single and/or in the double mutant, 20 common expression patterns were observed and divided into the six different types: buffering; suppression; quantitative buffering; quantitative suppression; masking; and inversion. Less frequent epistatic patterns were categorized as miscellaneous and excluded from downstream analysis.

### Functional enrichment analyses

For functional enrichment analyses, a hypergeometric testing procedure was performed using Gene Ontology (GO) biological process (BP) annotations [56] as obtained from the Saccharomyces Cerevisiae Database [57]. The background population was set to 6,359 (the number of genes annotated in GO) and *P* values were corrected for multiple testing using Bonferroni.

### GSTF promoter affinity scores calculated from *in vitro* data

GSTF promoter affinity scores were calculated from [27, 28]. First, signal intensities and enrichment scores for GSTFs that have been measured *in vitro* on multiple protein binding microarrays were averaged within each of the two datasets. The affinity by which a GSTF binds to the promoter of a potential target gene was then estimated by adding up signal intensities for each DNA 8-mer sequence, with an enrichment score greater than or equal to 0.45, within 600 base pairs upstream of the translation start site. Last, the resulting promoter affinity profile of one GSTF to all possible promoters was z-transformed to correct for experimental variation.

### Overlap between expression changes and DNA binding

For each of the exemplified GSTF pairs, genes with expression levels that changed dependently (all genes *i* where ε_*txpn,XYi*_ > log_2_(1.5)) or independently (all genes *i* where ε_*txpn,XYi*_ < log_2_(1.5)) of the respective genetic interaction were first sorted into different sets depending on the observed expression patterns. Overlap between genes in a given set and genes whose promoter is known to be bound by the respective GSTFs *in vivo* [26] (selected parameters are *P* = 0.005, no conservation restriction) was evaluated by Fisher’s exact test. Furthermore, a Mann–Whitney test was applied to test whether genes in a given set exhibit higher GSTF promoter affinity scores *in vitro* (calculated from [27, 28], as described above) than all other genes. Resulting *P* values were corrected for multiple testing using Benjamini-Hochberg; adjusted *P* values below 0.05 were considered significant.

### Availability of supporting data

The dataset supporting the results of this article is available in the ArrayExpress repository, E-MTAB-1385, http://www.ebi.ac.uk/arrayexpress/experiments/E-MTAB-1385/, as well as in the GEO repository, GSE42536, http://www.ncbi.nlm.nih.gov/geo/query/acc.cgi?acc=GSE42536. The data is also available as flat-file or in TreeView format from http://www.holstegelab.nl/publications/GSTF_geneticinteractions.

## Additional files

Additional file 1:
**List of putative GSTFs and strains used.** (XLSX 27 kb) Additional file 2:
**Replicate growth-based genetic interactions.** (**A**) Replicate fitness values derived from growth in liquid culture. Values are expressed as growth rates, relative to WT (RGR). GSTF single mutants are depicted as open circles, double mutants as solid circles. (**B**) Genetic interaction scores derived from growth on agar plates [11] (YEPD medium, horizontal) versus genetic interaction scores derived from growth in liquid culture (SC medium, vertical, this study). (PDF 248 kb) Additional file 3:
**Contribution of selection criteria to genetic interaction types.** (**A**) Hierarchical clustering of GSTF pairs selected on growth-based genetic interaction scores, represented as in Fig. 3. Clustering was performed on the epistatic effects. GSTF pairs marked with a solid circle were also selected based on similarity in DNA binding. Colored branches depict example groups described in the text. (**B**) Hierarchical clustering of GSTF pairs selected based on similarity in DNA binding, represented as in A. GSTF pairs marked with a solid circle also exhibit a genetic interaction as derived by growth on agar plates [11]. (PDF 339 kb) Additional file 4:
**Genetic interaction scores between GSTF pairs.** (XLSX 17 kb)

## Abbreviations

ERAD: endoplasmic reticulum-associated degradation
FC: fold change
GSTF: gene-specific transcription factor
RGR: relative growth rate
SC: synthetic complete
WT: wildtype
YEPD: yeast extract peptone dextrose
