# Supplementary figures and images for: A high-resolution gene expression atlas of epistasis between gene-specific transcription factors exposes potential mechanisms for genetic interactions
Source: BMC Biol. 2015 Dec 23;13:112. doi: 10.1186/s12915-015-0222-5 (PMC4690272; doi:10.1186/s12915-015-0222-5)

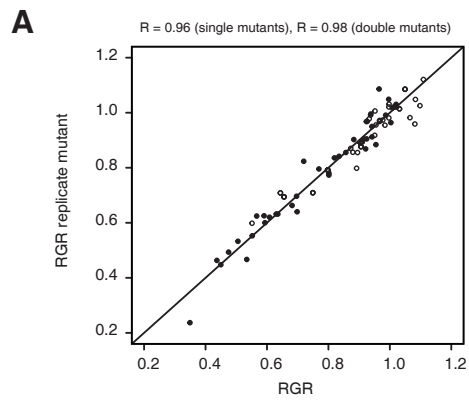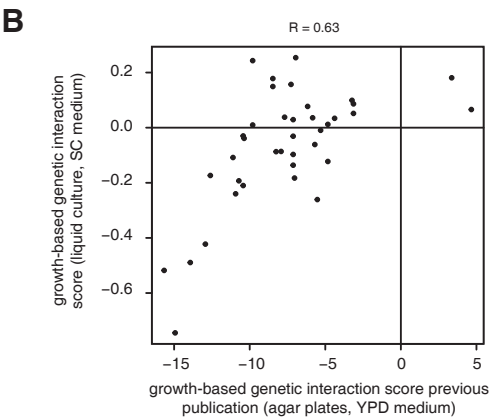

Supplement: Additional file 2: — Replicate growth-based genetic interactions. (A) Replicate fitness values derived from growth in liquid culture. Values are expressed as growth rates, relative to WT (RGR). GSTF single mutants are depicted as open circles, double mutants as solid circles. (B) Genetic interaction scores derived from growth on agar plates [11] (YEPD medium, horizontal) versus genetic interaction scores derived from growth in liquid culture (SC medium, vertical, this study). (PDF 248 kb) [file 12915_2015_222_MOESM2_ESM.pdf]

A

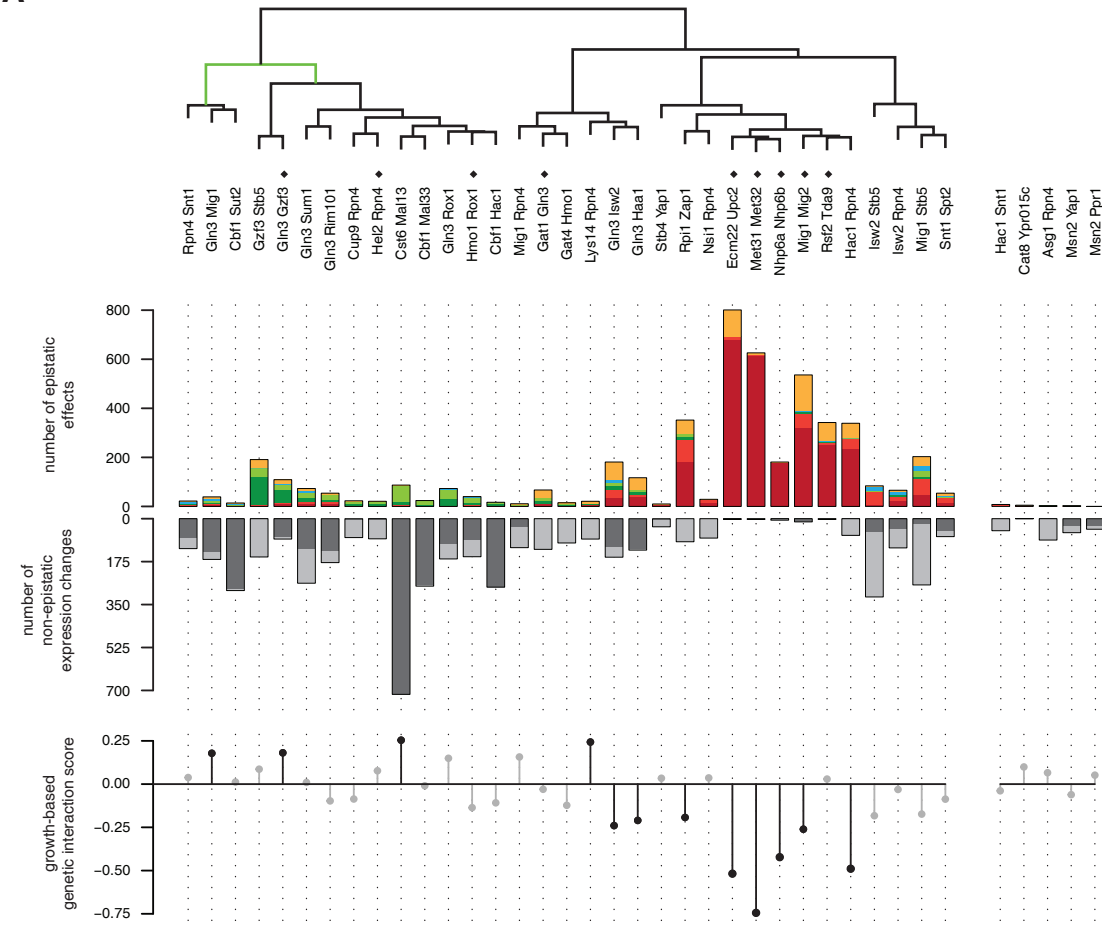

B

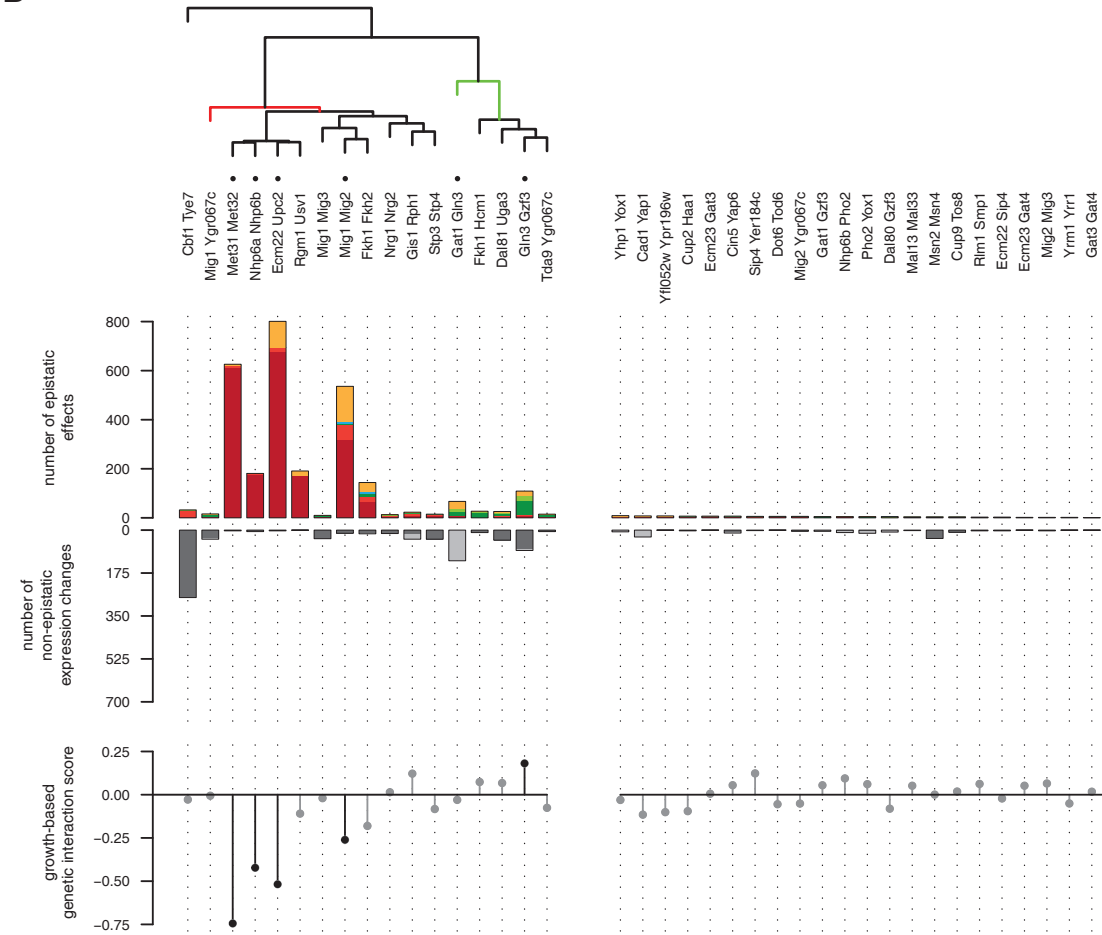

Supplement: Additional file 3: — Contribution of selection criteria to genetic interaction types. (A) Hierarchical clustering of GSTF pairs selected on growth-based genetic interaction scores, represented as in Fig. 3. Clustering was performed on the epistatic effects. GSTF pairs marked with a solid circle were also selected based on similarity in DNA binding. Colored branches depict example groups described in the text. (B) Hierarchical clustering of GSTF pairs selected based on similarity in DNA binding, represented as in A. GSTF pairs marked with a solid circle also exhibit a genetic interaction as derived by growth on agar plates [11]. (PDF 339 kb) [file 12915_2015_222_MOESM3_ESM.pdf]
